# Enterotropism of highly pathogenic avian influenza virus H5N8 from the 2016/2017 epidemic in some wild bird species

**DOI:** 10.1186/s13567-020-00841-6

**Published:** 2020-09-14

**Authors:** Valentina Caliendo, Lonneke Leijten, Lineke Begeman, Marjolein J. Poen, Ron A. M. Fouchier, Nancy Beerens, Thijs Kuiken

**Affiliations:** 1 Department of Viroscience, Erasmus Medical Center, Rotterdam, The Netherlands; 2 Department of Virology, Wageningen Bioveterinary Research, Lelystad, The Netherlands

**Keywords:** avian influenza, H5N8, H5N1, wild birds, tropism, virus histochemistry, immunohistochemistry, pathology, low pathogenic, highly pathogenic

## Abstract

In 2016/2017, H5N8 highly pathogenic avian influenza (HPAI) virus of the Goose/Guangdong lineage spread from Asia to Europe, causing the biggest and most widespread HPAI epidemic on record in wild and domestic birds in Europe. We hypothesized that the wide dissemination of the 2016 H5N8 virus resulted at least partly from a change in tissue tropism from the respiratory tract, as in older HPAIV viruses, to the intestinal tract, as in low pathogenic avian influenza (LPAI) viruses, allowing more efficient faecal-oral transmission. Therefore, we determined the tissue tropism and associated lesions in wild birds found dead during the 2016 H5N8 epidemic, as well as the pattern of attachment of 2016 H5N8 virus to respiratory and intestinal tissues of four key wild duck species. We found that, out of 39 H5N8-infected wild birds of 12 species, four species expressed virus antigen in both respiratory and intestinal epithelium, one species only in respiratory epithelium, and one species only in intestinal epithelium. Virus antigen expression was association with inflammation and necrosis in multiple tissues. The level of attachment to wild duck intestinal epithelia of 2016 H5N8 virus was comparable to that of LPAI H4N5 virus, and higher than that of 2005 H5N1 virus for two of the four duck species and chicken tested. Overall, these results indicate that 2016 H5N8 may have acquired a similar enterotropism to LPAI viruses, without having lost the respirotropism of older HPAI viruses of the Goose/Guangdong lineage. The increased enterotropism of 2016 H5N8 implies that this virus had an increased chance to persist long term in the wild waterbird reservoir.

## Introduction

Avian influenza causes major economic damage to the poultry industry, as well as welfare issues to the poultry involved. For example, the global highly pathogenic avian influenza (HPAI) virus epidemic of the subtype H5N8 in 2014/2015 led to the death or culling of over 50 million birds in seven countries in Asia, Europe, and North America [1–3]. In addition, the ability of HPAI viruses to cross the species barrier and cause severe disease in humans, other mammals, and wild birds poses a more general threat to human and animal health [4, 5].

The Goose/Guangdong lineage of H5 HPAI virus (originating from H5N1 HPAI virus A/Goose/Guangdong/1/1996) has persisted in poultry populations in parts of South-East Asia at least since 2003, and has circulated between poultry and wild birds, allowing continual virus evolution as well as reassortment of H5 HPAI virus with other avian influenza (AI) viruses [1, 3]. Descendants of the HPAI H5 Goose/Guangdong lineage are able to survive long enough in migrating wild birds to spread from South-East Asia as far as West Europe, North America, and—perhaps—South Africa [2]. The adaptation of HPAI virus to wild birds provides an additional route of virus incursion into poultry holdings, and expands the geographic range over which HPAI virus poses a threat to human and animal health [1].

Low pathogenic avian influenza (LPAI) viruses are endemic in wild birds of the orders *Anseriformes* and *Charadriiformes*. LPAI viruses replicate in intestinal epithelial cells and are excreted mainly from the cloaca [6–10]. These enterotropic viruses are transmitted by the faecal-oral route, including via the water bodies on which these birds reside [8]. In contrast, the H5 HPAI viruses that spread via migratory birds and caused epidemics in 2005/2006 and 2014/2015 were respirotropic and were excreted mainly from the pharynx. Specifically, experimentally infected ducks of several species showed evidence of virus replication in epithelial cells of the respiratory tract, but not of the intestinal tract [11–15]. The scant cloacal shedding detected in some of those birds was attributed to virus replication in the liver, pancreas, or both, which are in contact with the intestinal lumen via bile and pancreatic ducts, respectively [11]. Since then, H5 HPAI viruses spread again from Asia to Europe in 2016/2017, causing the biggest and most widespread epidemic on record in wild and domestic birds in Europe [3, 16].

It is unknown whether the H5 HPAI viruses from 2016/2017 have become better adapted to replication in and transmission among wild birds, and so allowed the virus to spread so widely in Europe and to infect so many wild birds. We hypothesized that the dissemination of H5 HPAI virus in wild birds in Europe in 2016/2017 resulted, at least in part, from a reversal of virus tropism from the respiratory tract to the intestinal tract, thus allowing more excretion from the cloaca and more efficient faecal-oral transmission via contaminated water. This would allow the phenotype of H5 HPAI virus to resemble that of LPAI virus, as a type of convergent evolution. To test this hypothesis, we reviewed literature of HPAI cases prior to 2016 to determine tissue tropism; determined tissue tropism and associated lesion in wild birds that died during 2016/2017 epidemic; and showed the pattern of virus attachment of 2016 H5N8 virus to respiratory and intestinal tissues of wild ducks, compared with older HPAI viruses.

## Materials and methods

### Study design

This study consisted of two parts. In the first part, we examined the carcasses of 39 wild birds that were found dead during the 2016/2017 H5N8 HPAI epidemic in The Netherlands and that tested positive for H5N8 HPAI virus (Table 1), in order to characterize the pathology and cell type tropism of this virus infection in different organs. We were particularly interested to determine whether the H5N8 HPAI virus had more tropism for the digestive tract of wild birds than that of HPAI viruses from previous epidemics, based on published accounts. For the literature review, we retrieved articles in English published between 2004 and 2018 from the PubMed database (http://www.ncbi.nlm.nih.gov/pubmed/). Key words used were “avian influenza”, “H5N1”, “H5N8”, “wild birds”, “immunohistochemistry” and “IHC”.

**Table 1:** Detection of HPAIV H5N8 in cloacal and oropharyngeal swabs from carcasses of wild ducks

Species	No. of birds	Positive RRT-PCR for H5N8 virus in:			
		Pooled CL and OP swabs	Separate CL and OP swabs	Separate CL swab only	Separate OP swab only
Tufted duck *Aythya fuligula*^a^	7	1	3	0	3
Common pochard *Aythya ferina*^a^	1	Nd	1	0	0
Great crested grebe *Podiceps cristatus*^a^	1	Nd	1	0	0
Eurasian teal *Anas crecca*^a^	1	Nd	1	0	0
Eurasian wigeon *Mareca penelope*^a^	10	3	7	0	0
Mallard *Anas platyrhynchos*	2	Nd	2	0	0
Duck (unspecified species)	10	10	Nd	Nd	Nd
Greylag goose *Anser anser*^a^	1	Nd	1	0	0
Great black backed gull *Larus marinus*^a^	1	Nd	1	0	0
Lesser black backed gull *Larus fuscus*	1	1	Nd	Nd	Nd
Black-headed gull *Chroicocephalus ridibundus*^a^	1	Nd	1	0	0
Eurasian buzzard *Buteo buteo*	2	1	0	0	1
Eurasian magpie *Pica pica*	1	1	Nd	Nd	Nd

*CL* cloacal, *OP* oropharyngeal, *Nd* not done.

^a^Previously reported in Poen et al. 2018 [7].

Our null hypothesis was that the 2005/2006 HPAI H5N1 and 2016/2017 HPAI H5N8 viruses have similar tropism for the intestinal tract of wild birds; the alternate hypothesis was that these two subtypes have a different tropism.

In the second part, we performed virus histochemical analysis of four avian influenza viruses in order to compare the pattern of attachment of these viruses in the respiratory and intestinal tracts of five bird species. We were particularly interested to determine whether the 2016/2017 H5N8 HPAI virus attached better to the digestive tract of four key wild duck species (Eurasian wigeon, *Mareca penelope*; mallard, *Anas platyrhynchos*; tufted duck, *Aythya fuligula*; common pochard, *Aythya ferina*) compared to the 2014/2015 HPAI H5N8 and 2005/2006 HPAI H5N1 viruses. We included a common LPAI virus as a representative virus with a clear tropism for the digestive tract of mallards, and included tissues of the chicken as a representative poultry species.

### Pathology and immunohistochemistry of naturally infected wild birds

The carcasses of 39 wild birds had been collected in the provinces of Flevoland, Gelderland, Noord and Zuid Holland (The Netherlands) in November and December 2016. All the birds tested positive for H5N8 2016 by real-time reverse-transcription PCR (RRT-PCR) assays in oropharyngeal and/or cloacal swabs as described previously [7].

The postmortem examinations and tissue sampling were performed according to a standard protocol. The following tissues, when available, were examined: brain, lungs, air sacs, pancreas, liver, stomachs (proventriculus and ventriculus), small intestine (jejunum, ileum), large intestine (cecum, colon), kidney, adrenal gland, spleen and heart. The tissues were fixed in 10% neutral-buffered formalin and embedded in paraffin. Tissues were sectioned at 3 µm and stained with hematoxylin and eosin for histopathological analysis or stained with a monoclonal antibody against nucleoprotein of influenza A virus for immunohistochemical detection of influenza viral antigen, as described previously [8].

### Virus histochemistry

The following four viral strains were used as input viruses: 2008 LPAIV H4N5 (A/Mallard/Netherlands/13/08), 2014 HPAIV H5N8 (A/Eurasian wigeon/Netherlands/emc-1/2014), 2016 HPAIV H5N8 (A/Eurasian wigeon/Netherlands/19/2016), 2005 HPAIV H5N1 (A/Turkey/Turkey/1/05). The viruses were individually passaged in Madin-Darby canine kidney (MDCK) cells. After 2–3 days, the supernatant was harvested and cleared of cell debris by low speed centrifugation for 20 min at 1455 × *g*. The viruses were individually concentrated by centrifugation of the cleared supernatants in filter tubes (Amicon Ultra-15 100 K filter-tubes, Millipore, UFC9100024, Darmstadt, Germany) for 40 min at 4000 × *g* at 4 °C. The concentrated virus was inactivated by dialysing against 0.1% formalin for 3 days at room temperature (RT). After inactivation, the virus solution was dialysed against phosphate-buffered saline solution (PBS) and complete inactivation was confirmed by passaging on MDCK cells. Virus was labelled by adding an equal volume of 0.1 mg/mL of fluoresceinisothiocyanate (FITC) (Sigma-Aldrich, Saint Louis, MO) in 0.5 M bicarbonate buffer (pH 9.5) for 1 h at RT while constantly stirring. Labelled virus was dialysed against PBS in order to lose all unbound FITC. The concentration of the different virus suspensions used for virus histochemistry was standardized at 50 hemagglutination units/100 μL (HAU) using hemagglutination assay.

Tissue sections of the following species were used: tufted duck (n = 3), common pochard (n = 2), Eurasian wigeon (n = 3), mallard (n = 3), and domestic chicken (n = 2). These tissues came from the Erasmus MC tissue bank, and were from healthy animals that showed no abnormalities or histological lesions. From the respiratory tract, tissues selected were trachea, primary bronchus, secondary bronchus, tertiary bronchus or parabronchus, air capillaries and air sacs. From the digestive tract of same birds, tissues selected were duodenum, jejunum, ileum and colon.

Three-μm-thick formalin-fixed paraffin-embedded sections of each tissue were deparaffinized in xylene and hydrated using graded alcohols and incubated overnight with FITC-labelled viruses at a concentration of 50 HAU/100 μL. To enable visualization by light microscopy, FITC was detected with a peroxidase-labeled rabbit anti-FITC antibody (DAKO, Glostrup, Denmark). The signal was amplified using a tyramide amplification system (Perkin-Elmer, Boston, MA). Peroxidase was revealed with 3-amino-9-ethylcarbazole (Sigma-Aldrich) resulting in a bright red precipitate. Tissues were counterstained with hematoxylin and embedded in glycerol-gelatin (Merck, Whitehouse Station, NJ). Omission of the FITC-labelled virus was used as a negative control.

The slides were assessed with light microscopy to estimate the abundance of viral attachment to epithelial cells and scored as follows: : attachment to < 1% of epithelial cells (−), attachment to ≥ 1 and < 10% of epithelial cells (±), attachment to ≥ 10% and < 50% of epithelial cells (+), and attachment to ≥ 50% of epithelial cells (++). Finally, the median score was determined for each species at the different anatomical sites. Sections were examined without knowledge of the identity of the birds.

## Results

### Influenza virus antigen expression and associated lesions in naturally infected wild birds

The 39 HPAI-virus-positive carcasses of 12 wild bird species, plus unspecified ducks (Table 1), were examined for influenza virus antigen expression. We were particularly interested in virus antigen expression in epithelial cells of digestive and respiratory tracts, because these correspond to virus excretion from cloaca and pharynx, respectively. Four wild bird species (Eurasian wigeon; tufted duck; black-headed gull, *Chroicocephalus ridubundus*; Eurasian magpie, *Pica pica*) expressed influenza virus antigen in epithelial cells of both digestive tract and respiratory tract (Table 2). One species (great black-backed gull, *Larus marinus*) and unspecified ducks expressed influenza virus antigen in epithelial cells of respiratory tract only, and one species (greylag goose, *Anser anser*) expressed influenza virus antigen in epithelial cells of digestive tract only. Besides epithelial cells, other cell types in digestive and respiratory tracts that expressed influenza virus antigen were endothelial cells and neurons. Besides tissues in digestive and respiratory tracts, tissues in other organs also expressed influenza virus antigen (see Additional file 1). The degree to which this occurred reflects the capacity of the virus to spread systemically.

**Table 2:** Expression of AIV antigen in cell types of different organs of the respiratory and gastro-intestinal tracts

Species	No of birds	Number of birds expressing influenza virus antigen in a cell type of an organ														
		Respiratory tract						Gastro-intestinal tract								
		Lung			Air sac			Proventriculus			Small intestine			Large intestine		
		EP	E	N	EP	E	N	EP	E	N	EP	E	N	EP	E	N
Tufted duck *Aythya fuligula*	7	1	4	0	0	1	0	1∞	2	2	1∞	4	1	0	4	0
Common pochard *Aythya farina*	1	0	0	0	0	0	0	0	0	0	0	0	1	0	0	0
Great crested grebe *Podiceps cristatus*	1	0	0	0	0	0	0	0	0	0	0	0	0	0	0	0
Eurasian teal *Anas crecca*	1	0	0	0	0	0	0	0	0	0	0	0	0	0	0	0
Eurasian wigeon *Mareca Penelope*	10	5	6	1	2	5	0	2∞	7	0	3∞*	8	1	1*	8	0
Mallard *Anas platyrhynchos*	2	0	0	0	1	0	0	0	1	0	1	1	1	0	1	0
Duck (unspecified species)	10	8	0	0	8	0	0	0	3	0	0	2	1	0	3	1
Greylag goose *Anser anser*	1	0	0	0	0	1	0	1	0	0	0	0	0	0	0	0
Great black backed gull *Larus marinus*	1	0	1	0	1	1	0	0	0	0	0	1	0	0	1	0
Lesser black backed gull *Larus fuscus*	1	1	0	0	1	0	0	0	0	0	0	0	0	0	0	0
Black-headed gull *Chroicocephalus ridibundus*	1	0	1	0	1	0	0	1	1	0	0	1	1	0	1	1
Eurasian buzzard *Buteo buteo*	2	0	1	0	0	0	0	0	0	0	0	0	0	0	0	0
Eurasian magpie *Pica pica*	1	1	0	0	1	0	0	1	0	0	1	0	0	1	0	0
Total	39	16	13	1	15	8	0	6	14	2	6	17	6	2	18	2

*Ep* epithelial cell, *E* endothelial cell, *N* neuron.

∞ One bird expressed influenza virus antigen in the epithelial cells of both proventriculus and small intestine.

*One bird expressed influenza virus antigen in the epithelial cells of both small and large intestine.

**Figure 1:**
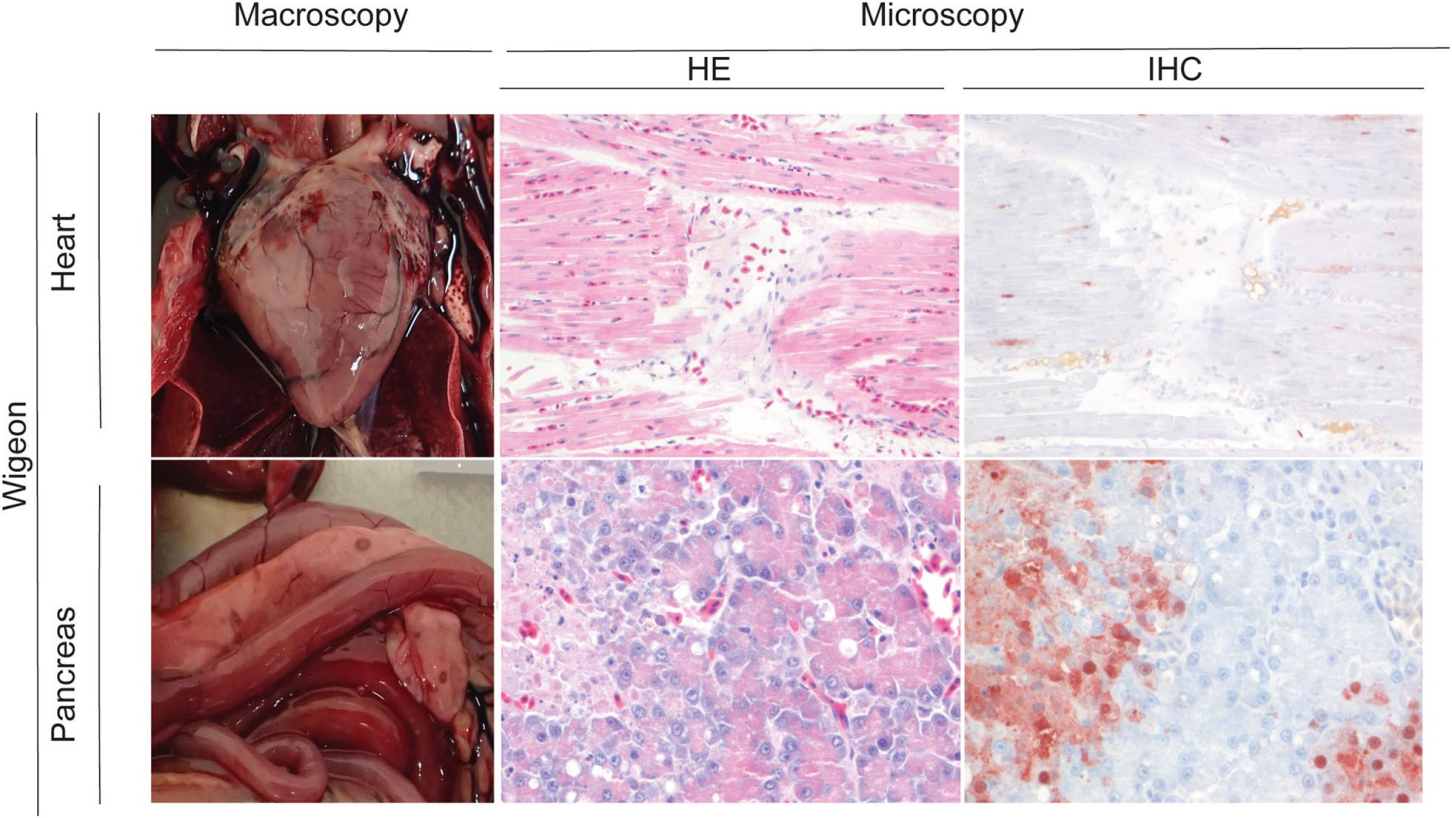
**Macroscopy, histological lesions and virus antigen expression in tissues of wild birds.**

**Figure 2:**
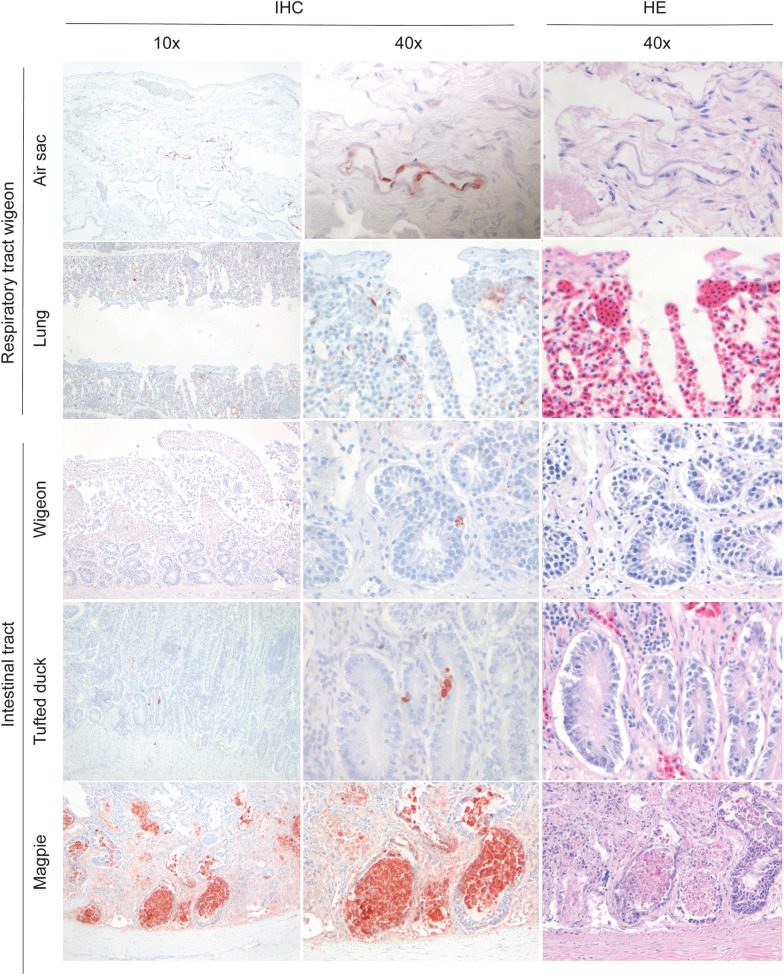
**Histological lesions and virus antigen expression in tissues of wild birds.**

Grossly, the main pathological changes consisted of multifocal necrosis in liver (7 birds) and pancreas (6 birds); sub-pericardial hemorrhages (10 birds); and multifocal pulmonary consolidation (6 birds) (see Additional file 2). Histologically, lesions were detected in the liver (16 birds), brain (12 birds), pancreas (8 birds), kidney (8 birds), lungs (8 birds), heart (8 birds), and intestine (7 birds). Lesions were characterized by multifocal necrosis in liver and pancreas; necrosis and inflammation in brain, intestine, and kidney; hemorrhages in lungs; and hemorrhages and necrosis in heart (Figures 1, 2; see Additional file 3).

### Pattern of virus attachment to epithelia of digestive and respiratory tracts

In intestinal epithelia, the level of attachment varied per virus and per host species (Table 3). In overall comparison among viruses, H5N1 had lower attachment to intestinal epithelia than 2014 H5N8, 2016 H5N8 and H4N5. In overall comparison among host species, virus attachment to intestinal epithelia was low in tufted duck and Eurasian pochard, intermediate in Eurasian wigeon, and high in chicken and mallard. For a given virus and a given host species, the level of attachment among different parts of the intestine (duodenum, jejunum, ileum, colon) was comparable.

**Table 3:** Pattern of attachment of avian influenza viruses to the epithelial cells of the intestinal tract

Species	Tissues	Avian influenza viruses			
		H4N5	2014 H5N8	2016 H5N8	H5N1
Mallard	Duodenum	++	+	++	++
	Jejunum	++	++	++	++
	Ileum	++	+	++	+
	Colon	++	+	++	+
Pochard	Duodenum	nd	±	±	±
	Jejunum	±	±	±	–
	Ileum	–	±	–	–
	Colon	±	±	–	–
Tufted Duck	Duodenum	±	±	±	±
	Jejunum	±	±	±	–
	Ileum	±	±	–	–
	Colon	±	±	–	±
Wigeon	Duodenum	+	±	±	±
	Jejunum	+	+	+	±
	Ileum	+	+	+	±
	Colon	+	+	+	±
Chicken	Duodenum	++	++	++	±
	Jejunum	++	++	++	±
	Ileum	++	++	++	±
	Colon	+	+	+	±

Mean abundance of attachment was scored as follows: attachment to < 1% of epithelial cells (-), attachment to ≥ 1 and < 10% of epithelial cells (±), attachment to ≥ 10% and < 50% of epithelial cells (+), and attachment to ≥ 50% of epithelial cells (++).

*nd* not detected.

The level of attachment of the different viruses to intestinal epithelia differed per host species. In the mallard, 2016 H5N8 had high attachment to intestinal epithelia, comparable to that of H4N5. It was higher than the level of attachment of H5N1 and 2014 H5N8, because of improved attachment to ileum and colon. In the Eurasian wigeon, both 2014 H5N8 and 2016 H5N8 had moderate attachment to intestinal epithelia, just lower than that of H4N5. These three viruses had higher attachment than that of H5N1, because of improved attachment to jejunum, ileum, and colon. In the tufted duck and Eurasian pochard, both 2014 H5N8 and 2016 H5N8 had low attachment to intestinal epithelia, comparable to that of H4N5. These three viruses had slightly higher attachment than that of H5N1, because of improved attachment to jejunum, ileum, and/or colon. In the chicken, both 2014 H5N8 and 2016 H5N8 had high attachment to intestinal epithelia, comparable to that of H4N5. These three viruses had markedly higher attachment than H5N1, because of improved attachment to all parts of the intestine.

In respiratory epithelia, the level of virus attachment differed per tissue, but not per virus or host species (Table 4). With a few exceptions, virus attachment to trachea, primary bronchi, secondary bronchi, and air sacs was high regardless of virus and host species. In contrast, virus attachment to parabronchi, atria, and air capillaries was low regardless of host species, except for the chicken, where attachment to parabronchi was moderate (2014 H5N8) to strong (other three viruses).

**Table 4:** Pattern of attachment of avian influenza viruses to the epithelial cells of the respiratory tract

Species	Tissues	Avian influenza viruses			
		H4N5	2014 H5N8	2016 H5N8	H5N1
Mallard	Trachea	++	++	++	++
	Primary bronchus	++	++	++	++
	Secondary bronchus	++	++	++	++
	Parabronchus atria	±	±	±	±
	Air capillaries	+	±	±	+
	Air sac	++	++	++	++
Pochard	Trachea	++	++	++	+
	Primary bronchus	++	++	++	+
	Secondary bronchus	++	++	++	++
	Parabronchus atria	±	±	±	±
	Air capillaries	±	±	±	+
	Air sac	++	++	++	++
Tufted Duck	Trachea	++	+	++	++
	Primary bronchus	++	++	++	++
	Secondary bronchus	++	++	++	++
	Parabronchus atria	±	±	±	+
	Air capillaries	+	±	±	+
	Air sac	++	+	++	++
Wigeon	Trachea	++	++	++	++
	Primary bronchus	++	++	++	++
	Secondary bronchus	++	++	++	++
	Parabronchus atria	±	±	±	±
	Air capillaries	±	±	±	+
	Air sac	++	++	++	++
Chicken	Trachea	++	++	++	++
	Primary bronchus	++	++	++	+
	Secondary bronchus	++	++	++	+
	Parabronchus atria	++	+	++	++
	Air capillaries	±	–	±	+
	Air sac	++	++	++	++

Mean abundance of attachment was scored as follows: attachment to < 1% of epithelial cells (-), attachment to ≥ 1 and < 10% of epithelial cells (±), attachment to ≥ 10% and < 50% of epithelial cells (+), and attachment to ≥ 50% of epithelial cells (++).

*nd* not detected.

A review of natural and experimental HPAI virus infections revealed 18 articles in first search. Of these 18 articles, 15 examined the gastro-intestinal tract but failed to detect HPAI virus antigen expression in gastro-intestinal epithelium. Of these 15 articles, 7 articles did not report any virus antigen expression at all in the gastro-intestinal tract, and 8 articles reported the presence of virus antigen expression in non-epithelial tissues: vascular endothelium or parasympathetic ganglia in the submucosal and muscular plexi of the gastro-intestinal tract. In the remaining three articles, influenza virus antigen expression [for, respectively, H5N1/2005; A/chicken/Vietnam/14/2005 (H5N1); A/swan/Germany/R65/06(H5N1)] was detected in the gastro-intestinal epithelium of three species: Eurasian magpie, Canada goose (*Branta canadensis*), and whooper swan (*Cygnus cygnus*) [17–35] (see Additional files 4, 5).

## Discussion

The null hypothesis that H5N1 and 2016 H5N8 have similar tropism for the intestinal tract was rejected based on both virus antigen expression and virus attachment studies. Based on virus antigen expression, intestinal epithelium expressed 2016 H5N8 antigen in four of twelve bird species (5 of 29 individual birds, excluding 10 unspecified ducks) that were naturally infected with 2016 H5N8, compared to the more limited virus antigen expression in the intestinal epithelium of wild birds infected with earlier viruses of the Goose/Guangdong lineage (this study). Based on virus attachment, 2016 H5N8 attached better than H5N1 to intestinal epithelium in three of four duck species tested. Based on these results, we accept the alternate hypothesis that 2016 H5N8 is more enterotropic than H5N1.

Enterotropism of HPAI virus is a novel phenomenon in wild birds. According to our review, infection of epithelial cells of the gastro-intestinal tract was reported in only three or four species (Eurasian magpie, Canada goose, and mute and/or whooper swan), none of them ducks. In contrast to HPAI virus, LPAI virus is well known to be enterotropic in wild birds. In fact, the epithelium of intestine and cloacal bursa were the only tissues that expressed LPAI virus antigen in naturally infected mallards and black-headed gulls [8, 9]. An important difference between the two pathotypes of AI virus is that LPAI virus infection is not known to cause any intestinal lesions in wild birds, while 2016 H5N8 infection caused marked necrosis and inflammation of the intestinal mucosa (this study) [10]. Two caveats regarding virus antigen detection in intestinal mucosa are that intestinal mucosa autolyses rapidly after death of a bird, making it more difficult to detect virus antigen, and that, historically, intestinal tissues have not been sampled extensively. To improve the sensitivity of influenza virus detection in intestinal mucosa, carcasses should be cooled to 4 °C, samples of intestine should be collected and fixed in formalin as soon as possible after death, and the intestinal mucosa should be sampled at multiple levels, from duodenum to colon. A useful technique to increase the amount of intestinal mucosa to be scanned for virus antigen expression is the so-called ‘Swiss role’ technique, which allowed a 7-cm-long segment of mallard intestine to be embedded in one paraffin block prior to making tissue sections [20].

In addition to gaining enterotropism, 2016 H5N8 retained respirotropism. Infection of the respiratory tract is considered to be the main source of HPAI virus excretion from the oropharynx in wild birds [11]. In the seven birds in our study that expressed influenza virus antigen in gastro-intestinal epithelium, five also expressed it in respiratory epithelium. This situation is intermediate between respirotropic H5N1 infection and enterotropic LPAI virus infection [9, 11].

Besides in respiratory tract and intestinal tract, 2016 H5N8 antigen also was expressed in other tissues, including in particular brain, liver, lung, heart, and pancreas. Infection in most of these tissues was associated with both necrosis and inflammation, and it is likely that these 2016 H5N8-associated lesions were fatal to the birds in our study. The character and severity of these lesions were similar to those caused by H5N1 infection, with the exception of intestinal lesions, which are not present in H5N1 infection [18]. However, this does not necessarily mean that 2016 H5N8 and 2005 H5N1 are comparable in virulence. It is very difficult to estimate the virulence of HPAI virus infection in wild birds from field data, since we do not know how many wild birds were infected, and which proportion of infected birds died. Therefore, the case fatality rate (proportion of infected individuals that died) is unknown both for H5N8 and 2005 H5N1.

Based on experimental infections in different species of ducks, 2014 H5N8 from the USA and from the Netherlands is less virulent than H5N1 [12, 15]. However, the more recent 2016 H5N8 shows an increased virulence compared to 2014 H5N8 and it is able to produce severe disease in waterfowl both in natural and experimental settings [16, 22]. The reasons that caused this increase in virulence are still unclear but, from a clinical and pathological point of view, this severity of the disease is likely due to a more systemic involvement.

From the results of this study, we can conclude that 2016 H5N8 has a tropism for both digestive tract and respiratory tract of at least four wild bird species (Eurasian wigeon, tufted duck, black-headed gull, Eurasian magpie) based on virus antigen detection in naturally infected birds. This means that 2016 H5N8 mirrors the enterotropism of LPAIV, without having lost the respirotropism of older viruses of the Goose/Guangdong lineage, like H5N1. How the H5 viruses of the Goose/Guangdong lineage will evolve in wild birds in future is not clear. One possibility is that they become completely enterotropic, like LPAIV in mallards and black-headed gulls. Another possibility is that they retain tropism for both digestive and respiratory tracts. It is conceivable that such a dual tropism provides maximum flexibility for the virus to adapt to multiple species of wild and domestic birds, depending on the ecological niche where the virus happens to be.

The implications of increased tropism for the digestive tract is that relatively more virus is excreted from the cloaca and contaminates the water bodies on which wild waterbirds reside. Indirect transmission via contaminated water, up to several weeks after the infected birds have left the water body, is a key factor for the maintenance of LPAI in wild waterbirds [23]. If indirect transmission via contaminated water becomes a major route of transmission for H5N8 or other viruses of the Goose/Guangdong lineage, they have an increased chance to persist indefinitely in the wild waterbird reservoir.

## Supplementary information

**Additional file 1.** Expression of avian influenza virus antigen in cell types of other organs than those of respiratory and gastro-intestinal tracts. Number of birds expressing influenza virus antigen in a cell type of an organ.

**Additional file 2.** Frequency and distribution of gross lesions associated with virus antigen expression in carcasses of wild birds. Number of birds with gross lesions in different organs.

**Additional file 3.** Frequency and distribution of histological lesions associated with virus antigen expression in carcasses of wild birds. Number of birds with histological lesions in different organs.

**Additional file 4.** Literature review of articles in English from the PubMed database. Literature reporting influenza virus antigen expression in the gastro-intestinal epithelium.

**Additional file 5.** Common and scientific name of additional bird species mentioned in Additional file 4. Common and scientific names of the birds mentioned.

## Data Availability

Not applicable.
