# Supplementary material for: Enterotropism of highly pathogenic avian influenza virus H5N8 from the 2016/2017 epidemic in some wild bird species
Source: Vet Res. 2020 Sep 14;51:117. doi: 10.1186/s13567-020-00841-6 (PMC7491185; doi:10.1186/s13567-020-00841-6)
Supplement: Supplementary file 1 — Additional file 1. Expression of avian influenza virus antigen in cell types of other organs than those of respiratory and gastro-intestinal tracts. Number of birds expressing influenza virus antigen in a cell type of an organ. [file 13567_2020_841_MOESM1_ESM.docx]

Additional file 1: Expression of avian influenza virus antigen in cell types of other organs than those of respiratory and gastro-intestinal tracts.

|  | Number of birds expressing influenza virus antigen in a cell type of an organ | | | | | | | | | | | | | | | | | |
| --- | --- | --- | --- | --- | --- | --- | --- | --- | --- | --- | --- | --- | --- | --- | --- | --- | --- | --- |
|  |  |  |  |  |  |  |  |  |  |  |  |  |  |  |  |  |  |  |
|  |  | Nervous system | | | | |  | Digestive system | | | | |  | Other systems | | | | |
|  |  | Brain | |  | Peripheral nerve | |  | Pancreas | |  | Liver | |  | Adrenal gland | |  | Heart | |
| Species | No of birds | E | N |  | E | N |  | E | EP |  | E | H |  | CC | N |  | E | M |
| Tufted duck  *Aythya fuligula* | 7 | 3 | 0 |  | 2 | 1 |  | 0 | 1 |  | 0 | 4 |  | 2 | 1 |  | 0 | 3 |
| Common pochard  *Aythya ferina* | 1 | 0 | 0 |  | 0 | 0 |  | 0 | 0 |  | 0 | 0 |  | 0 | 0 |  | 0 | 0 |
| Great crested grebe  *Podiceps cristatus* | 1 | 0 | 0 |  | 0 | 0 |  | 0 | 0 |  | 0 | 0 |  | 0 | 0 |  | 0 | 0 |
| Eurasian teal  *Anas crecca* | 1 | 0 | 0 |  | 0 | 0 |  | 0 | 0 |  | 1 | 0 |  | 0 | 0 |  | 0 | 0 |
| Eurasian wigeon  *Mareca penelope* | 10 | 4 | 3 |  | 4 | 0 |  | 3 | 7 |  | 4 | 7 |  | 1 | 0 |  | 4 | 3 |
| Mallard  *Anas platyrhynchos* | 2 | 0 | 1 |  | 0 | 1 |  | 0 | 0 |  | 0 | 2 |  | 0 | 1 |  | 0 | 0 |
| Duck  (unspecified species) | 10 | 0 | 0 |  | 0 | 0 |  | 0 | 0 |  | 0 | 0 |  | 0 | 0 |  | 0 | 0 |
| Greylag goose  *Anser anser* | 1 | 1 | 0 |  | 1 | 0 |  | 0 | 1 |  | 0 | 1 |  | 0 | 1 |  | 0 | 1 |
| Great black backed gull  *Larus marinus* | 1 | 0 | 0 |  | 0 | 0 |  | 0 | 0 |  | 0 | 0 |  | 0 | 0 |  | 0 | 0 |
| Lesser black backed gull  *Larus fuscus* | 1 | 1 | 0 |  | 0 | 0 |  | 0 | 0 |  | 0 | 0 |  | 1 | 0 |  | 0 | 0 |
| Black-headed gull  *Chroicocephalus ridibundus* | 1 | 0 | 1 |  | 1 | 0 |  | 1 | 1 |  | 0 | 1 |  | 0 | 0 |  | 0 | 1 |
| Eurasian buzzard  *Buteo buteo* | 2 | 0 | 1 |  | 0 | 0 |  | 0 | 1 |  | 0 | 1 |  | 0 | 0 |  | 0 | 1 |
| Eurasian magpie  *Pica pica* | 1 | 1 | 1 |  | 0 | 0 |  | 0 | 1 |  | 0 | 1 |  | 0 | 0 |  | 0 | 0 |

Ep, epithelial cell; E, endothelial cell; N, neuron; H, hepatocyte; CC, chromaffin cell; M, myocytes.
