# Supplementary material for: Enterotropism of highly pathogenic avian influenza virus H5N8 from the 2016/2017 epidemic in some wild bird species
Source: Vet Res. 2020 Sep 14;51:117. doi: 10.1186/s13567-020-00841-6 (PMC7491185; doi:10.1186/s13567-020-00841-6)
Supplement: Supplementary file 2 — Additional file 2. Frequency and distribution of gross lesions associated with virus antigen expression in carcasses of wild birds. Number of birds with gross lesions in different organs. [file 13567_2020_841_MOESM2_ESM.docx]

Additional file 2: Frequency and distribution of gross lesions associated with virus antigen expression in carcasses of wild birds.

|  |  | No. of birds with gross lesions in the: | | | | | | | | | |
| --- | --- | --- | --- | --- | --- | --- | --- | --- | --- | --- | --- |
| Species | No of birds | Gross lesions (Organs)? | Air sac‡ | Heartπ | Intestine | Kidney | Liver# | Lung§ | Pancreas¶ | Proventriculus∞ | Unknown |
| Tufted duck  *Aythya fuligula* | 7 | Y (H,L,Lu,P) | 0 | 3 | 0 | 0 | 1 | 3 | 2 | 0 | 4 |
| Common pochard  *Aythya ferina* | 1 | N | 0 | 0 | 0 | 0 | 0 | 0 | 0 | 0 |  |
| Great crested grebe  *Podiceps cristatus* | 1 | N | 0 | 0 | 0 | 0 | 0 | 0 | 0 | 0 |  |
| Eurasian teal  *Anas crecca* | 1 | N | 0 | 0 | 0 | 0 | 0 | 0 | 0 | 0 |  |
| Eurasian wigeon  *Mareca penelope* | 10 | Y(H,L, Lu, P,Pr) | 0 | 4 | 0 | 0 | 5 | 3 | 4 | 2 | 3 |
| Mallard  *Anas platyrhynchos* | 2 | Y(A,H) | 1 | 1 | 0 | 0 | 0 | 0 | 0 | 0 |  |
| Duck  (unspecified species) | 10 | UK | NR | NR | NR | NR | NR | NR | NR | NR | 10 |
| Greylag goose  *Anser anser* | 1 | N | 0 | 0 | 0 | 0 | 0 | 0 | 0 | 0 |  |
| Great black backed gull  *Larus marinus* | 1 | N | 0 | 0 | 0 | 0 | 0 | 0 | 0 | 0 |  |
| Lesser black backed gull  *Larus fuscus* | 1 | UK | NR | NR | NR | NR | NR | NR | NR | NR | 1 |
| Black-headed gull  *Chroicocephalus ridibundus* | 1 | Y(H,I) | 1 | 1 | 0 | 0 | 0 | 0 | 0 | 0 |  |
| Eurasian buzzard  *Buteo buteo* | 2 | Y(H,L) | 0 | 1 | 0 | 0 | 1 | 0 | 0 | 0 | 1 |
| Eurasian magpie  *Pica pica* | 1 | UK | NR | NR | NR | NR | NR | NR | NR | NR | 1 |

A,air sac; H, heart; I, intestine; K, kidney; L, liver; Lu, lung; N, no; NR, not recorded P, pancreas; Pr, proventriculus; UK, unknown; Y, yes

‡Airsacculitis, characterized by mild, multifocal to diffuse opacity and thickening of the air sacs.

πSubpericardial hemorrhage

#Diffuse hepatic necrosis.

§Multifocal pulmonary consolidation.

¶Multifocal pancreatic necrosis, consisting of red or gray foci of 1–4 mm in diameter affecting up to 25% of the pancreas.

∞Hemorrhages
