# Supplementary material for: Enterotropism of highly pathogenic avian influenza virus H5N8 from the 2016/2017 epidemic in some wild bird species
Source: Vet Res. 2020 Sep 14;51:117. doi: 10.1186/s13567-020-00841-6 (PMC7491185; doi:10.1186/s13567-020-00841-6)
Supplement: Supplementary file 3 — Additional file 3. Frequency and distribution of histological lesions associated with virus antigen expression in carcasses of wild birds. Number of birds with histological lesions in different organs. [file 13567_2020_841_MOESM3_ESM.docx]

Additional file 3: Frequency and distribution of histological lesions associated with virus antigen expression in carcasses of wild birds.

|  |  |  | No. of birds with histological lesions in the: | | | | | | | | | |
| --- | --- | --- | --- | --- | --- | --- | --- | --- | --- | --- | --- | --- |
| Species | No of birds | Histological lesions (Organs)? | Air sac‡ | Brainꭥ | Heartπ | Intestineꞷ | Kidneyꟸ | Liver# | Lung§ | Pancreas¶ | Proventriculus∞ | Unknown |
| Tufted duck  *Aythya fuligula* | 7 | Y(A,B,H,I,K,L,Lu,P,Pr) | 2 | 3 | 3 | 3 | 1 | 3 | 3 | 2 | 1 | 4 |
| Common pochard  *Aythya ferina* | 1 | Y(L) | 0 | 0 | 0 | 0 | 0 | 1 | 0 | 0 | 0 |  |
| Great crested grebe  *Podiceps cristatus* | 1 | N | 0 | 0 | 0 | 0 | 0 | 0 | 0 | 0 | 0 |  |
| Eurasian teal  *Anas crecca* | 1 | Y(L) | 0 | 0 | 0 | 0 | 0 | 1 | 0 | 0 | 0 |  |
| Eurasian wigeon  *Mareca penelope* | 10 | Y(B,H,I,K,L, Lu, P,Pr) | 0 | 5 | 4 | 2 | 4 | 6 | 4 | 4 | 1 | 3 |
| Mallard  *Anas platyrhynchos* | 2 | Y(B,L) | 0 | 1 | 0 | 0 | 0 | 2 | 0 | 0 | 0 |  |
| Duck  (unspecified species) | 10 | UK | Na | na | na | na | Na | na | na | na | na | 10 |
| Greylag goose  *Anser anser* | 1 | Y(B,L) | 0 | 1 | 0 | 0 | 0 | 1 | 0 | 0 | 0 |  |
| Great black backed gull  *Larus marinus* | 1 | Y(L,Lu) | 0 | 0 | 0 | 0 | 0 | 1 | 1 | 0 | 0 |  |
| Lesser black backed gull  *Larus fuscus* | 1 | UK | Na | na | na | na | Na | na | na | na | na | 1 |
| Black-headed gull  *Chroicocephalus ridibundus* | 1 | Y(B,I,L) | 0 | 1 | 0 | 1 | 0 | 1 | 0 | 0 | 0 |  |
| Eurasian buzzard  *Buteo buteo* | 2 | Y(B,H,L,P) | 0 | 1 | 1 | 0 | 0 | 1 | 0 | 1 | 0 |  |
| Eurasian magpie  *Pica pica* | 1 | Y(I,P,Pr) | 0 | 0 | 0 | 1 | 0 | 0 | 0 | 1 | 1 |  |

A,air sac; B, brain; H, heart; I, intestine; K, kidney; L, liver; Lu, lung; N, no; P, pancreas; Pr, proventriculus; na, not available; Y, yes

‡ Airsacculitis, characterized by multifocal aggregates of lymphocytes and plasma cells in the interstitium . ꭥ Necrotizing encephalitis with gliosis.

Π Hyperaemia, multifocal hemorrhages and necrosis of cardiomyocytes

ꞷ Necrosis and inflammation of the intestinal mucosa.

ꟸ Necrosis and interstitial nephritis.

# Multifocal necrosis of hepatocytes.

§ Hyperemia, edema and hemorrhage.

¶ Moderate to severe, multifocal to confluent acinar necrosis

∞ Inflammatory lymphoplasmacellular infiltration.
