# Supplementary material for: Enterotropism of highly pathogenic avian influenza virus H5N8 from the 2016/2017 epidemic in some wild bird species
Source: Vet Res. 2020 Sep 14;51:117. doi: 10.1186/s13567-020-00841-6 (PMC7491185; doi:10.1186/s13567-020-00841-6)
Supplement: Supplementary file 4 — Additional file 4. Literature review of articles in English from the PubMed database. Literature reporting influenza virus antigen expression in the gastro-intestinal epithelium. [file 13567_2020_841_MOESM4_ESM.docx]

Additional file 4 : Literature review of articles in English from the PubMed database.

| **Antigen in epithelium of digestive tract present** | **Species** | **Infection type** | **Virus** | **Location of viral antigen in digestive tract** | **Cell infection recorded** | **References** |
| --- | --- | --- | --- | --- | --- | --- |
| Y | Magpie | N | H5N1 | Intestine | Intestinal epithelium | Kwon 2005  [17] |
| Y | Canada goose | E | A/  chicken/Vietnam/14/2005 (H5N1) | Proventriculus, small  intestine and cecum | Epithelium, parasympathetic ganglia and mesenteric plexi, occasional scattered smooth muscle and vascular  endothelial cell | Pasick 2007  [18] |
| Y | Whooper swan | N | A/swan/Germany/R65/06(H5N1) | Proventriculus | Epithelium | Teifke 2007  [19] |
| n | Bar-headed goose,  Canada goose | N | H5N1 | None |  | Ellis 2004  [24] |
| n | Eastern Zhejiang white geese | E | A/Bar-headedGoose/Qinghai/0510/05 (H5N1) | None |  | Zhou 2006  [25] |
| n | Mallard, northern  pintail, common teal, redhead, wood duck, laughing gulls | E | A/Whooper  Swan/Mongolia/244/05 (H5N1), A/Duck Meat/Anyang/01 (H5N1) | Small intestines | Parasympathetic ganglia in the submucosal and muscular plexus | Brown 2006  [26] |
| n | Pekin duck | E | A/Thailand PB/6231/04(H5N1) | Proventriculus |  | Pantin-Jacwood 2007  [27] |
| n | Pekin duck | E | A/Crow/Thailand/04(H5N1) | Proventriculus, intestine |  | Pantin-Jacwood 2007  [27] |
| n | Pekin duck | E | A/Egret/HK/7572/02(H5N1) | Proventriculus, intestine |  | Pantin-Jacwood 2007  [27] |
| n | Call duck | E | A/chicken/Yamaguchi/7/04(H5N1) | None |  | Yamamoto 2007  [28] |
| n | Tufted duck | E | A/turkey/Turkey/1/05(H5N1) | None |  | Londt 2008  [29] |
| n | Mute swan | E | A/Cygnuscygnus/Germany/R65/2006(H5N1) | Proventriculus, intestine | Vascular endothelium | Kalthoff 2008  [30] |
| n | Tufted ducks, Eurasian pochards, mallards common teals, Eurasian wigeons, gadwalls | E | A/turkey/Turkey/1/2005 (H5N1) | None |  | Keawcharoen 2008  [11] |
| n | Tufted ducks | N | H5N1 | Proventriculus |  | Brojer 2009  [31] |
| n | Canada goose | E | A/chicken/Vietnam/14/05(H5N1) | Proventriculus, duodenum, ceca |  | Neufeld 2009  [32] |
| n | Mute swan, greylag goose, mandarin duck | E | A/chicken/Korea/IS/06(H5N1) | Small and large intestine | Vascular endothelium | Kwon 2010  [33] |
| n | Pekin duck | E | A/duck/Sleman/BBVW-59832226/2007(H5N1) | None |  | Wibawa 2013  [34] |
| n | Coot | N | H5N8 2014 | Intestine |  | Kim 2014  [35] |
| n | Eurasian wigeon, common pochard mallard, common teal | E | A/chicken/Netherlands/emc-3/2014 (H5N8) | None |  | Van de Brand 2018  [12] |
| n | Pekin ducks, muscovy duck | E | DE14¬H5N8A | Proventriculus |  | Grund 2018  [22] |
| n | Pekin ducks, muscovy duck | E | DE16¬H5N8B | Proventriculus, duodenum |  | Grund 2018  [22] |

Y yes, n no, N natural, E experimental,
