# Supplementary material for: Enterotropism of highly pathogenic avian influenza virus H5N8 from the 2016/2017 epidemic in some wild bird species
Source: Vet Res. 2020 Sep 14;51:117. doi: 10.1186/s13567-020-00841-6 (PMC7491185; doi:10.1186/s13567-020-00841-6)
Supplement: Supplementary file 5 — Additional file 5. Common and scientific name of additional bird species mentioned in Additional file 4. Common and scientific names of the birds mentioned. [file 13567_2020_841_MOESM5_ESM.docx]

Additional file 5: Common and scientific name of additional bird species mentioned in Additional file 4

| **Common name** | **Scientific name** |
| --- | --- |
| Bar-headed goose | *Anser indicus* |
| Black swan | *Cygnus atratus* |
| Call duck | *Anas platyrhynchos* |
| Canada goose | *Branta canadensis* |
| Coot | *Fulica atra* |
| Laughing gull | *Larus atricilla* |
| Mandarin duck | *Aix galericulata* |
| Muscovy duck | *Cairina moschata* |
| Mute swan | *Cygnus olor* |
| Northern pintail | *Anas acuta* |
| Pekin duck | *Anas platyrhynchos domesticus* |
| Redhead | *Aythya americana* |
| Trumpeter swan | *Cygnus buccinator* |
| Whooper swan | *Cygnus cygnus* |
| Wood duck | *Aix sponsa* |
